# Clinical outcomes of early-stage triple-negative breast cancer after neoadjuvant chemotherapy according to HER2-low status☆

**DOI:** 10.1016/j.esmoop.2024.103973

**Published:** 2024-11-04

**Authors:** A.S. Raghavendra, D.B. Zakon, Q. Jin, A. Strahan, M. Grimm, M.E. Hughes, M. Cherian, J. Vincuilla, T. Parker, P. Tarantino, E.A. Mittendorf, T.A. King, V. Valero, D. Tripathy, S.M. Tolaney, N. Tayob, N.U. Lin, D.G. Stover, C.H. Barcenas, A.C. Garrido-Castro

**Affiliations:** 1 Department of Breast Medical Oncology, The University of Texas MD Anderson Cancer Center, Houston, USA; 2 Department of Data Science, Dana-Farber Cancer Institute, Boston, USA; 3 The Ohio State University Comprehensive Cancer Center, Columbus, USA; 4 Department of Medical Oncology, Dana-Farber Cancer Institute, Boston, USA; 5 Breast Oncology Program, Dana-Farber Brigham Cancer Center, Boston, USA; 6 Harvard Medical School, Boston, USA; 7 Division of Breast Surgery, Department of Surgery, Brigham and Women’s Hospital, Boston, USA

**Keywords:** HER2 low, early stage, TNBC, pCR, survival, breast cancer

## Abstract

**Background:**

The impact of human epidermal growth factor receptor 2 (HER2) expression determined by immunohistochemistry (IHC) on outcomes in early-stage triple-negative breast cancer (eTNBC) is unclear. Using a large, multi-institutional cohort, we evaluated outcomes by HER2 IHC status in patients with eTNBC who received neoadjuvant therapy (NAT).

**Patients and methods:**

Patients with stage I-III TNBC who received NAT and underwent surgery from January 2016 to June 2019 were identified from three databases. HER2 expression was defined as low (IHC1+ or 2+/FISH not amplified) or HER2 IHC score 0 by local testing at diagnosis. Pathological complete response (pCR) rates were compared using logistic regression adjusted for multiple factors. Survival outcomes were estimated using Kaplan–Meier and Cox proportional hazards models.

**Results:**

Among 977 consecutive patients, 388 (39.7%) had HER2-low and 589 (60.3%) had HER2 IHC score 0 tumors. Median age at eTNBC diagnosis was 50.3 years (range 21.0-83.4 years). At baseline, clinical nodal positivity rate was significantly higher in HER2-low (55.0%) versus HER2 IHC score 0 tumors (46.6%) (*P* = 0.011); pCR rates were similar (32.0% versus 32.6%; adjusted *P* = 0.924). At a median follow-up of 3.5 years, recurrence-free survival (RFS) did not vary significantly between HER2-low versus HER2 IHC score 0 among patients with pCR (adjusted *P* = 0.368) or residual disease (RD) after NAT (adjusted *P* = 0.573). Distant RFS and overall survival (OS) did not differ by HER2 category for patients with pCR [distant RFS (DRFS), adjusted *P* = 0.509; OS, adjusted *P* = 0.514] or RD (DRFS, adjusted *P* = 0.812; OS, *P* = 0.285). Discordance of tumor HER2 status was seen in 31.1% of HER2 IHC score 0 cases, with HER2 expression observed post-treatment; 34.8% of HER2-low cases showed discordance, with absent HER2 expression in RD.

**Conclusions:**

In this large cohort of patients with eTNBC treated with NAT, HER2-low status was not associated with pCR or survival after adjusting for clinical factors. The discordance in HER2 IHC pre- and post-NAT likely reflects challenges in HER2 quantification and heterogeneity.

## Highlights

• HER2-low eTNBC showed no pCR or survival benefit over HER2 IHC score 0.
• RFS did not significantly differ between HER2 IHC categories after NAT.
• HER2-low tumors had higher nodal positivity (55%) than HER2 IHC score 0 (46.6%); *P* = 0.011.
• Post-treatment HER2 IHC status changed in 31.1% of HER2 IHC score 0 and 34.8% of HER2-low cases.
• HER2 IHC status discordance underscores quantification and heterogeneity challenges.

## Introduction

Human epidermal growth factor receptor 2 (HER2) status in patients with breast cancer has been categorized as positive or negative according to immunohistochemistry (IHC) and FISH following the American Society of Clinical Oncology/College of American Pathologists (ASCO/CAP) guidelines.^1^ However, a major shift in the consideration of HER2 status for breast cancer management has taken place given that breast tumors classified as HER2-negative frequently have low levels of HER2 protein expression. Specifically, cases with IHC values of 1+ or 2+ and accompanying non-amplified FISH results are now categorized as HER2-low.^2^ With the development of antibody–drug conjugates (ADCs) such as fam–trastuzumab deruxtecan (T-DXd), distinguishing HER2-low from HER2 IHC score 0 tumors has become clinically relevant.^3^

The prevalence rate for HER2-low breast cancer ranges from 45% to 55% of all breast cancers, with a higher prevalence in patients with estrogen receptor (ER)-positive cancer than in those with triple-negative breast cancer (TNBC)^4^ (45.9% versus 29.4%).^5^ Conversely, approximately one-quarter of HER2-low cancers are concurrently ER-negative and progesterone receptor (PR)-negative, therefore defined as TNBC. Tumors with 1%-10% of cells staining positive for ER are categorized as ‘ER low positive’ as defined by the ASCO/CAP guidelines^6^ and similar outcomes to ER-negative disease have been reported.^7^

Much of the recent data obtained by researchers seeking to understand the unique features of HER2-low status derive from comparative analyses with HER2 IHC score 0 tumors to investigate variations in genomics, response to treatment, and prognosis. In general, researchers have found few significant differences in outcomes between HER2-low and HER2 IHC score 0 tumors, including pathological complete response (pCR) to neoadjuvant therapy (NAT) and overall survival (OS).^8^, ^9^, ^10^ However, comparison of HER2-low and HER2 IHC score 0 status in patients with early-stage, node-negative breast cancers has suggested that patients with HER2-low status have longer disease-free survival (DFS) and OS.^11^ In another study, HER2-low TNBC was associated with better breast cancer-specific survival than HER2 IHC score 0 TNBC, suggesting a potential interaction between HER2 expression levels and prognosis for TNBC.^12^

The standard treatment for most patients diagnosed with early-stage TNBC (eTNBC) includes NAT.^13^^,^^14^ The use of NAT for eTNBC is integral to the evaluation of prognosis and tumor response. NAT can lead to downstaging of tumor size and nodal involvement and facilitate the use of pCR as a prognostic and treatment decision tool, with pCR being a favorable indicator of improved long-term outcomes. Examination of long-term outcomes of eTNBC, especially in patients with pCR versus those with residual disease (RD), is critical to understanding the effectiveness of NAT. There is a paucity of data comparing outcomes in patients with HER2-low and HER2 IHC score 0 tumors with TNBC, specifically among patients who receive NAT. A subset study from the National Cancer Database demonstrated that the pCR rates were 30.2% for HER2-low TNBC and 33.4% for HER2 IHC score 0 TNBC^15^; however, only OS was reported, without data on breast cancer recurrence, and other studies to date have not definitively addressed this critical question.

Here, we sought to understand outcomes in eTNBC according to HER2 status in patients who received NAT in a large, multi-institutional cohort. Focusing on the TNBC subtype is key, given variable responses to NAT and long-term outcomes in early-stage HER2-negative breast cancers. Understanding the potential impact of HER2-low status on treatment response in TNBC is therefore important in formulating newer approaches to optimize therapy. The hypothesis for this retrospective study was that HER2-low status, as determined by IHC, would be associated with distinct outcomes in patients with eTNBC who received NAT.

## Patients and methods

### Population

Patients diagnosed with stage I-III TNBC (defined as ER and PR <10%, including hormone receptor-low and -negative tumors) who received NAT and underwent surgery from 1 January 2016 to 30 June 2019 were identified in three institutional prospective clinical databases at The University of Texas MD Anderson Cancer Center, Dana-Farber Cancer Institute, and The Ohio State University. Specifically, the databases were searched to identify patients who were at least 18 years old and had been diagnosed with eTNBC [HER2-negative as defined by ASCO/CAP guidelines (i.e. IHC 0, 1+, or 2+ with FISH results negative for HER2 amplification)]. This study was approved by the institutional review boards of all three institutions with a waiver of consent.

### Clinicopathological parameters

Detailed patient demographic, clinical, and pathological data were collected, including age at diagnosis, race, germline *BRCA1/2* (gBRCA) status, initial tumor stage, clinical nodal status, and recorded neoadjuvant and adjuvant treatments. HER2 expression was defined as low (IHC 1+ or 2+/FISH not amplified) or HER2 IHC score 0 according to clinical testing at primary tumor diagnosis.

Cancer-related outcomes including response to NAT (pCR versus the presence of RD) and the disease and vital status at last follow-up were assessed. pCR was defined as no invasive RD in the breast or axilla, allowing for the presence of ductal carcinoma *in situ* (ypT0/ypTis ypN0). Residual cancer burden (RCB) is a quantitative measure used to assess the amount of disease remaining after NAT. RCB is estimated from primary tissue and considers various factors such as tumor size, lymph node involvement, and the extent of residual cancer cells in the breast and lymph nodes after treatment.^16^ We collected and determined RCB to provide a standardized way to evaluate the response to treatment and predict prognosis. Recurrence-free survival (RFS) was defined as the duration from primary surgery to development of recurrence at a local or distant site or death due to any cause, whichever occurred first. Distant RFS (DRFS) was defined as the duration from primary surgery to development of metastasis at a distant site or death owing to any cause, whichever occurred first. OS was defined as the duration from primary surgery to death due to any cause. Patients who were still alive at the time of their last follow-up visit were censored in the analysis.

### Statistical analysis

Multivariable logistic regression analysis was used to compare pCR rates for HER2 IHC score 0 and HER2-low TNBC cases with adjustment for age at diagnosis, race, anatomic clinical stage, gBRCA status, histology, hormone receptor status, and receipt of both anthracycline- and taxane-based NAT. Contrasts in patient and tumor characteristics were evaluated using Pearson’s chi-square tests. RFS, DRFS, and OS durations were estimated using the Kaplan–Meier method. A multivariable Cox proportional hazards model was used to estimate adjusted hazard ratios (HRs). The threshold for significance based on the *P* value was set at <0.05.

## Results

### Clinicopathological characteristics

A total of 977 consecutive patients with eTNBC were identified from the three participating institutions for inclusion in analyses. The median patient age at eTNBC diagnosis was 50.3 years (range 21.0-83.4 years). Demographic and clinicopathological characteristics of the overall cohort and according to HER2 status are displayed in Table 1 and Supplementary Table S1, available at https://doi.org/10.1016/j.esmoop.2024.103973. There were 388 (39.7%) patients with HER2-low tumors and 589 (60.3%) with HER2 IHC score 0 tumors at diagnosis; 174 (17.8%) patients had hormone receptor-low tumors, and 142 (14.5%) had known gBRCA mutations. The majority of patients (*n* = 791; 80.9%) received multi-agent anthracycline and taxane (with or without platinum) as NAT. None of the patients received pembrolizumab. There were no significant differences between HER2-low and HER2 IHC score 0 groups with respect to age, race, gBRCA status, histology, hormone receptor status (low versus negative), clinical tumor size, or type of NAT (Table 1). The clinical nodal positivity rate was numerically higher in the HER2-low group (55.0%) than in the HER2 IHC score 0 group (46.6%; *P* = 0.011) and, by extension, distribution of clinical stage (I versus II versus III) followed a similar pattern with a greater proportion of stage III breast cancers among the HER2-low group (*P* = 0.023).

**Table 1:** Patient characteristics

	*n* (%)			
Characteristic	Overall (*n* = 977)	HER2-low (*n* = 388)	HER2 IHC score 0 (*n* = 589)	*P* value
Database site				0.091
MD Anderson	553 (56.6)	224 (57.7)	329 (55.9)	
DFCI	267 (27.3)	93 (24.0)	174 (29.5)	
OSU	157 (16.1)	71 (18.3)	86 (14.6)	
Age at TNBC diagnosis, years				0.105
Mean (±SD)	50.5 ± 12.6	51.3 ± 12.5	50.0 ± 12.7	
Median (range)	50.3 (21.0-83.4)	50.5 (21.3-83.3)	49.8 (21.0-83.4)	
Race				0.136
White	746 (76.4)	306 (78.9)	440 (74.7)	
Black	132 (13.5)	47 (12.1)	85 (14.4)	
Asian or Pacific Islander	48 (4.9)	13 (3.4)	35 (5.9)	
Other	39 (4.0)	19 (4.9)	20 (3.4)	
Unknown/not reported	12 (1.2)	3 (0.8)	9 (1.5)	
Overall gBRCA status				0.071 (*BRCA1*/*2* versus other versus unknown versus no mutation)
No mutation	597 (61.1)	229 (59.0)	368 (62.5)	
*BRCA1*/*2*	142 (14.5)	50 (12.9)	92 (15.6)	
Not tested	124 (12.7)	52 (13.4)	72 (12.2)	
Tested (N/A)	73 (7.5)	38 (9.8)	35 (5.9)	
Other gene mutation	41 (4.2)	19 (4.9)	22 (3.7)	
AJCC clinical stage (7th edition)				0.023 (I versus II versus III)
IA	106 (10.8)	40 (10.3)	66 (11.2)	
IB	9 (0.9)	4 (1.0)	5 (0.8)	
IIA	357 (36.5)	126 (32.5)	231 (39.2)	
IIB	218 (22.3)	85 (21.9)	133 (22.6)	
IIIA	100 (10.2)	44 (11.3)	56 (9.5)	
IIIB	93 (9.5)	41 (10.6)	52 (8.8)	
IIIC	94 (9.6)	48 (12.4)	46 (7.8)	
Final surgical procedure				0.112 (BCT versus mastectomy)
Breast-conserving therapy	371 (38.0)	134 (34.5)	237 (40.2)	
Unilateral mastectomy	283 (29.0)	121 (31.2)	162 (27.5)	
Bilateral mastectomy	228 (23.3)	87 (22.4)	141 (23.9)	
Mastectomy	87 (8.9)	40 (10.3)	47 (8.0)	
Axillary surgery only	8 (0.8)	6 (1.5)	2 (0.3)	
Axillary diagnostic procedure				0.057 (SLNB only versus ALND only versus both)
SLNB only	437 (44.7)	161 (41.5)	276 (46.9)	
ALND only	317 (32.4)	136 (35.1)	181 (30.7)	
Both	60 (6.1)	19 (4.9)	41 (7.0)	
None	6 (0.6)	1 (0.3)	5 (0.8)	
Unknown	157 (16.1)	71 (18.3)	86 (14.6)	
Type of histology				0.293
Invasive ductal	909 (93.0)	356 (91.8)	553 (93.9)	
Invasive lobular	7 (0.7)	4 (1.0)	3 (0.5)	
Mixed (invasive ductal and lobular)	13 (1.3)	8 (2.1)	5 (0.8)	
Other	47 (4.8)	19 (4.9)	28 (4.8)	
Unknown	1 (0.1)	1 (0.3)	0	
Residual nodal disease				0.854
Yes	333 (34.1)	136 (35.1)	197 (33.4)	
No	578 (59.2)	227 (58.5)	351 (59.6)	
Unknown	66 (6.8)	25 (6.4)	41 (7.0)	
Hormone receptor				0.657
Negative	803 (82.2)	322 (83.0)	481 (81.7)	
Low positive	174 (17.8)	66 (17.0)	108 (18.3)	
HER2 by IHC				—
0	589 (60.3)	0	589 (100)	
1+	266 (27.2)	266 (68.6)	0	
2+	122 (12.5)	122 (31.4)	0	
Neoadjuvant chemotherapy				—
Anthracycline + taxane	611 (62.5)	246 (63.4)	365 (62.0)	
Anthracycline + taxane + platinum	180 (18.4)	78 (20.1)	102 (17.3)	
Taxane alone	53 (5.4)	21 (5.4)	32 (5.4)	
Anthracycline alone	41 (4.2)	11 (2.8)	30 (5.1)	
Taxane + platinum	39 (4.0)	16 (4.1)	23 (3.9)	
Platinum alone	28 (2.9)	7 (1.8)	21 (3.6)	
Unknown	25 (2.6)	9 (2.3)	16 (2.7)	

AJCC, American Joint Committee on Cancer; ALND, axillary lymph node dissection; BCT, breast-conserving therapy; DFCI, Dana-Farber Cancer Institute; gBRCA, germline *BRCA1/2*; HER2, human epidermal growth factor receptor 2; IHC, immunohistochemistry; N+, node positive; N/A, not available; OSU, The Ohio State University; SD, standard deviation; SLNB, sentinel lymph node biopsy; TNBC, triple-negative breast cancer.

### HER2-low status and response to NAT among eTNBC

There were no significant differences in pCR rate between the HER2-low and HER2 IHC score 0 groups (32.0% versus 32.6%; *P* = 0.890) as shown in Table 2. Adjusting for age at diagnosis, race, anatomic clinical stage, gBRCA status, histology, hormone receptor status, and anthracycline- and taxane-based NAT, we observed no significant differences in pCR between the HER2-low and HER2 IHC score 0 groups {adjusted odds ratio: 0.99 [95% confidence interval (CI) 0.74-1.32, *P* = 0.924]} (Supplementary Table S2, available at https://doi.org/10.1016/j.esmoop.2024.103973). The odds ratio for the pCR rate suggested that HER2 status (HER2-low versus HER2 IHC score 0) does not significantly impact the likelihood of pCR in patients with eTNBC even after accounting for various clinical factors known to influence treatment response. Supplementary Table S3, available at https://doi.org/10.1016/j.esmoop.2024.103973, shows findings of pCR rates suggesting that in the patients who received anthracycline- and taxane-based NAT, the pCR rate did not differ markedly for HER2-low and HER2 IHC score 0 tumors, implying that HER2-low versus HER2 IHC score 0 status was not a significant predictor of response in this group. For the subset of patients who had germline testing, HER2 expression levels (HER2-low versus HER2 IHC score 0) did not significantly differ between gBRCA mutation and wild-type groups, and the HER2 status conversion rates post-NAT were similar between both groups. Germline *BRCA1/2* mutation carriers were significantly more likely to experience pCR than patients with germline *BRCA1/2* wild-type status (44.4% versus 30.1%; *P* = 0.012) (Supplementary Table S4, available at https://doi.org/10.1016/j.esmoop.2024.103973), confirming the expected influence of gBRCA status on NAT response.

**Table 2:** Comparison of pCR rates in patients with HER2-low and HER2 IHC score 0 eTNBC

	*n* (%)			
	Total (*n* = 977)	HER2-low (*n* = 388)	HER2 IHC score 0 (*n* = 589)	*P* value
pCR	316 (32.3)	124 (32.0)	192 (32.6)	0.890
RD	661 (67.7)	264 (68.0)	397 (67.4)	
RCB 0	316 (32.3)	124 (32.0)	192 (32.6)	
RCB I	94 (9.6)	39 (10.1)	55 (9.3)	
RCB II	304 (31.2)	107 (27.6)	197 (33.4)	
RCB III	146 (15.0)	66 (17.0)	80 (13.6)	
Unknown/not documented	117 (12.0)	52 (13.4)	65 (11.0)	

eTNBC, early-stage triple-negative breast cancer; HER2, human epidermal growth factor receptor 2; IHC, immunohistochemistry; pCR, pathological complete response; RCB, residual cancer burden; RD, residual disease.

### Long-term outcomes for HER2-low versus HER2 IHC score 0 tumors

**Figure 1:**
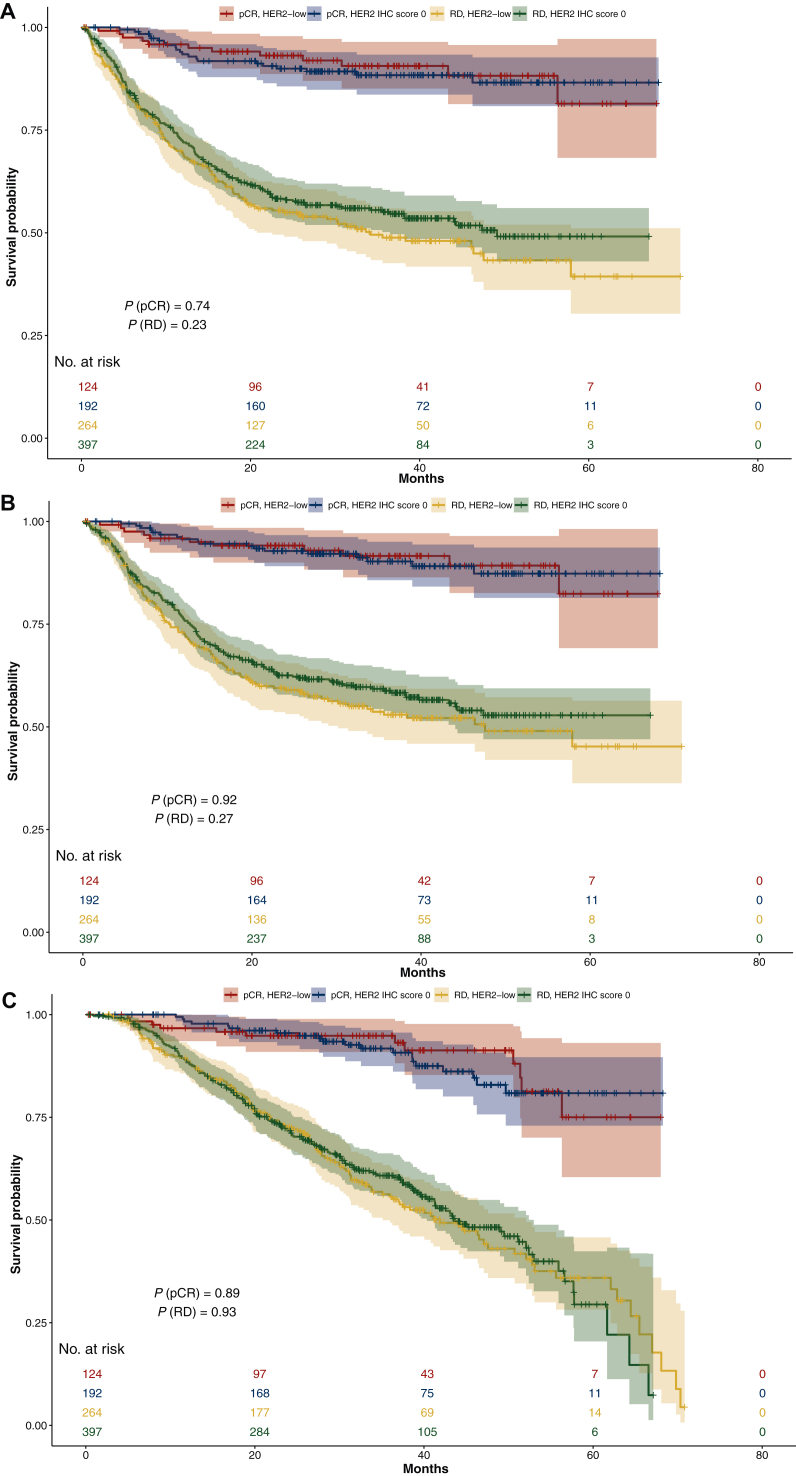
Kaplan–Meier plots of (A) RFS, (B) DRFS, and (C) OS according to HER2-low versus HER2 IHC score 0 in patients with pCR or RD after NAT. DRFS, distant RFS; HER2, human epidermal growth factor receptor 2; IHC, immunohistochemistry; NAT, neoadjuvant therapy; OS, overall survival; pCR, pathological complete response; RD, residual disease; RFS, recurrence-free survival.

At a median follow-up of 3.5 years, RFS did not differ significantly between HER2-low and HER2 IHC score 0 groups among patients experiencing pCR (adjusted *P* = 0.368) or those with RD after NAT (adjusted *P* = 0.573). Similarly, DRFS did not differ according to HER2 category for patients achieving pCR (adjusted *P* = 0.509) or RD (adjusted *P* = 0.812), nor did OS for patients achieving pCR (adjusted *P* = 0.514) or RD (adjusted *P* = 0.285) (Table 3, Figure 1).

**Table 3:** RFS, DRFS, and OS analysis by HER2 expression status

	pCR				Non-pCR			
	Survival probabilities (95% CI)				Survival probabilities (95% CI)			
Year	HER2-low (*n* = 124)	HER2 IHC score 0 (*n* = 192)	Adjusted HR (HER2- low versus HER2 IHC score 0)	*P* value	HER2-low (*n* = 264)	HER2 IHC score 0 (*n* = 397)	Adjusted HR (HER2- low versus HER2 IHC score 0)	*P* value
**RFS (years)**								
1	0.959 (0.924-0.995)	0.935 (0.899-0.971)	0.71 (0.34-1.49)	0.368	0.691 (0.637-0.751)	0.723 (0.679-0.769)	1.07 (0.85-1.35)	0.573
2	0.931 (0.887-0.979)	0.900 (0.857-0.945)			0.550 (0.491-0.616)	0.583 (0.535-0.635)		
3	0.906 (0.851-0.965)	0.884 (0.836-0.934)			0.488 (0.426-0.559)	0.551 (0.502-0.604)		
**DRFS (years)**								
1	0.959 (0.924-0.995)	0.957 (0.928-0.987)	0.77 (0.35-1.69)	0.509	0.726 (0.673-0.783)	0.761 (0.719-0.804)	1.03 (0.81-1.32)	0.812
2	0.941 (0.900-0.984)	0.928 (0.891-0.967)			0.590 (0.531-0.655)	0.625 (0.578-0.676)		
3	0.916 (0.864-0.971)	0.903 (0.857-0.950)			0.530 (0.468-0.600)	0.588 (0.539-0.641)		
**OS (years)**								
1	0.967 (0.935-0.999)	0.983 (0.965-1.000)	0.75 (0.32-1.78)	0.514	0.901 (0.865-0.939)	0.899 (0.869-0.929)	0.86 (0.65-1.14)	0.285
2	0.949 (0.909-0.990)	0.960 (0.932-0.990)			0.759 (0.707-0.815)	0.762 (0.719-0.807)		
3	0.949 (0.909-0.990)	0.937 (0.899-0.976)			0.649 (0.587-0.718)	0.666 (0.617-0.719)		

Adjusted HRs were calculated using a multivariable Cox proportional hazards model with adjustment for age at diagnosis, race, anatomic clinical stage, gBRCA status, histology, HR status, and anthracycline- and taxane-based NAT.

CI, confidence interval; DRFS, distant RFS; gBRCA, germline *BRCA1/2*; HER2, human epidermal growth factor receptor 2; HR, hazard ratio; IHC, immunohistochemistry; NAT, neoadjuvant therapy; OS, overall survival; pCR, pathological complete response; RFS, recurrence-free survival.

### Discordance in HER2 status from diagnosis to residual disease after NAT

**Figure 2:**
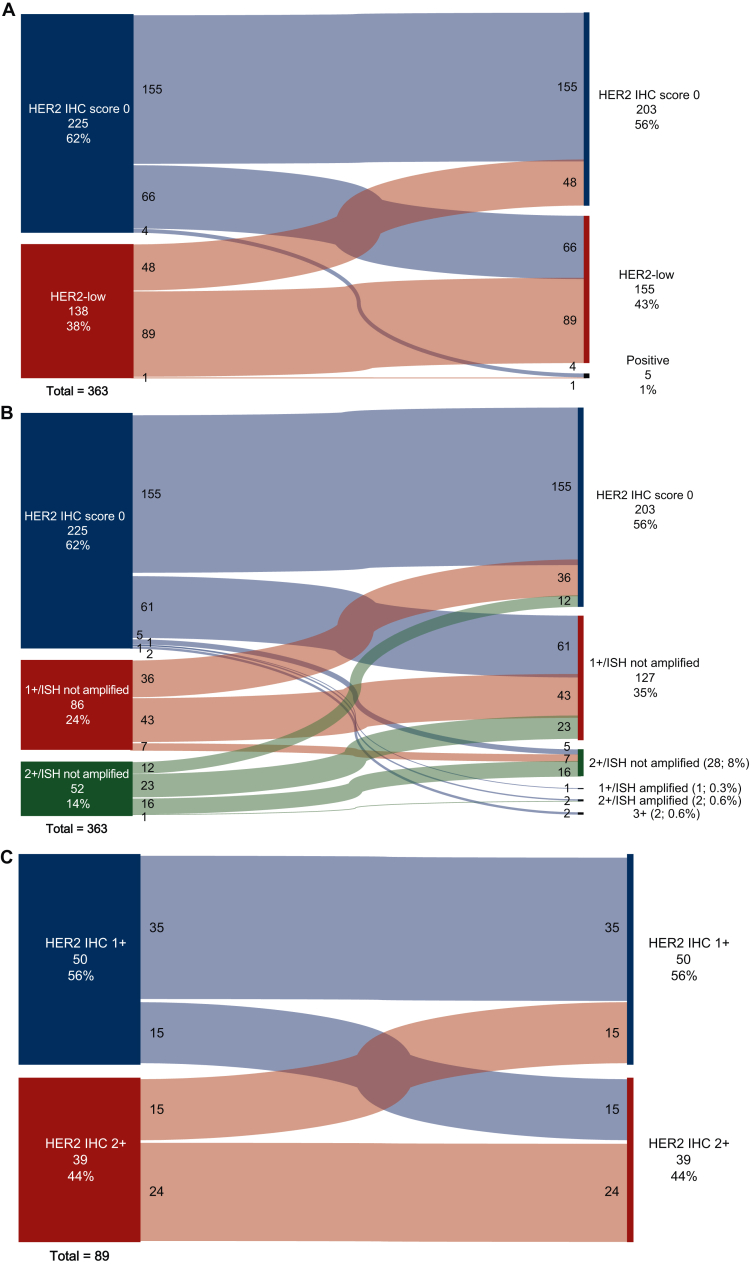
**Change in HER2 score from TNBC diagnosis to surgery post-NAT**. (A) Change in HER2-low versus HER2 IHC score 0.^a^ (B) Change in HER2 IHC (0 versus 1+ versus 2+).^b^ (C) Change in HER2-low IHC (1+ versus 2+).^c^ HER2, human epidermal growth factor receptor 2; IHC, immunohistochemistry; ISH, *in situ* hybridization; NAT, neoadjuvant therapy; RD, residual disease; TNBC, triple-negative breast cancer. ^a^Among patients with RD after NAT who had HER2 status available from both diagnostic biopsy and surgical samples (*n* = 363). ^b^Among patients with RD after NAT who had HER2 status available from both diagnostic biopsy and surgical samples (*n* = 363). ^c^Among patients with HER2-low tumors at both diagnosis and surgery post-NAT (*n* = 89).

Among 363 patients with RD after NAT and available HER2 IHC results, 244 (67.2%) had concordant HER2 status between the diagnostic biopsy and post-NAT surgical specimen (89 HER2-low at diagnosis and surgery; 155 HER2 IHC score 0 at diagnosis and surgery). Seventy of 225 (31.1%) patients with HER2 IHC score 0 tumors at diagnosis had HER2 expression after NAT, and 48 of 138 (34.8%) patients with HER2-low tumors had residual HER2 IHC score 0 tumors after NAT (Figure 2A).

When analyzing specific changes in HER2 expression, 43 patients (11.8%) with HER2 1+ tumors remained 1+ at RD, and 43 patients (11.8%) shifted to either HER2 IHC score 0 or 2+; 16 patients (4.4%) with HER2 2+ tumors at diagnosis remained 2+/FISH not amplified at RD, 35 patients (9.6%) shifted to HER2 IHC score 0 or 1+, and 1 patient (0.3%) had 2+/FISH-amplified RD; 155 patients (42.7%) with HER2 IHC score 0 tumors at diagnosis remained HER2 IHC score 0 at RD, while 66 patients (18.2%) transitioned to HER2-low (1+ or 2+), and 1.2% (4 patients) moved to 3+ or FISH-amplified HER2 status (Figure 2B).

Among 89 patients with HER2-low tumors at both diagnosis and RD post-NAT, most patients retained their HER2 IHC score post-NAT—35/89 (39.3%) IHC 1+ and 24/89 (27.0%) IHC 2+. The notable changes from 1+ to 2+ and from 2+ to 1+ (both at 16.9%) indicate variability in HER2 expression in response to NAT (Figure 2C).

## Discussion

In the present study, we explored the clinical and pathological attributes of a significant proportion of patients (39.7%) with HER2-low tumors in a large, well-annotated eTNBC population. Despite the differences in anatomic clinical staging and clinical nodal positivity between HER2-low and HER2 IHC score 0 tumors, with higher rates of nodal involvement in patients with HER2-low tumors, our findings demonstrated that the pathologic response to NAT did not significantly differ between the HER2-low and HER2 IHC score 0 groups. In addition, we evaluated the impact of HER2-low status on outcomes by assessing long-term survival and found no significant associations of HER2-low status with RFS, DRFS, or OS.

Our findings regarding pCR rates in patients with HER2-low TNBC are consistent with several previous studies that demonstrated similar pCR rates for HER2-low and HER2 IHC score 0 eTNBC.^17^, ^18^, ^19^ This finding is particularly noteworthy because it suggests that eTNBC HER2-low status does not confer differential sensitivity to NAT from that for eTNBC HER2 IHC score 0 tumors. This contrasts with what might be expected, given the prognostic impact of HER2 status defined by formal criteria.^2^^,^^20^ When considering the type of chemotherapy received, regimens with taxanes reduced the relapse rate more in HER2 2+ and 3+ cases compared to HER2 1+ and HER2 IHC score 0 cases. Anthracyclines alone also decreased the relapse rate in the HER2 3+ subgroup.^21^

Regarding long-term outcomes, we found no significant differences in RFS, DRFS, or OS between the eTNBC HER2-low and HER2 IHC score 0 groups regardless of whether they experienced pCR or RD after NAT. These results suggest that HER2 status at initial diagnosis is not a strong prognostic indicator for patients with TNBC who receive NAT, and that pCR is a favorable prognostic factor irrespective of HER2 expression levels. It is also worth mentioning the limitations of using pCR as a surrogate endpoint for long-term survival as it may not fully capture the heterogeneity of TNBC biology and response to treatment. While pCR is associated with favorable DFS, a subset of patients with RD after NAT still have favorable long-term outcomes, without experiencing breast cancer recurrence. In our study, the similar pCR rates observed between HER2-low and HER2 IHC score 0 eTNBC suggest that conventional chemotherapy regimens have comparable effectiveness for both subtypes. However, the lack of a significant difference in pCR rates does not preclude the possibility of distinct molecular characteristics and therapeutic vulnerabilities within the HER2-low eTNBC subtype.

A meta-analysis^22^ investigating survival outcomes following NAT revealed that patients with HER2-low tumors had a significantly lower pCR rate compared to those with HER2 IHC score 0, irrespective of hormone receptor status, but no significant differences in DFS and OS were observed between HER2-low and HER2 IHC score 0 in the TNBC group. In contrast, a large meta-analysis^23^ that compared clinical outcomes in patients with HER2-low versus HER2 IHC score 0 tumors found no difference in pCR rates or DFS between HER2 IHC groups in patients with TNBC, although OS was significantly longer in those with HER2-low tumors. Another recent study^24^ provided a comprehensive examination of the correlation between HER2 expression levels and survival outcomes in patients with eTNBC. The findings suggested that, while pCR rates did not differ considerably between HER2 IHC score 0 and HER2-low tumors, HER2-low status was associated with improved breast cancer-specific survival but not OS. This underscores the potential prognostic impact of HER2-low status on eTNBC. Further research is necessary to explore these possible distinctions and their clinical implications.

To ensure adequate follow-up for long-term outcomes, our study was conducted in a patient population who received NAT before the United States Food and Drug Administration (FDA) approval of pembrolizumab for high-risk, early-stage TNBC in July 2021. Future studies investigating the effectiveness of immunotherapy specifically in HER2-low TNBC are warranted to elucidate its potential role in the management of this tumor subtype. Furthermore, HER2-low status may guide the incorporation of novel therapies such as ADCs and poly (ADP-ribose) polymerase inhibitors into the treatment paradigm for eTNBC. Preclinical and clinical studies^25^, ^26^, ^27^, ^28^ have yielded promising results with these agents for HER2-low breast cancer, potentially offering more effective and less toxic treatment options than traditional chemotherapy. Trastuzumab deruxtecan (T-DXd) is currently FDA approved for the treatment of advanced HER2-low breast cancer after prior chemotherapy based on results from the DESTINY-Breast04 trial.^3^ In the recently reported DESTINY-Breast06 trial,^29^ T-DXd also demonstrated improved antitumor activity compared to chemotherapy of physician’s choice in patients with hormone receptor-positive/HER2-low metastatic breast cancer following endocrine therapy. In a prespecified exploratory analysis in patients with HER2-ultralow tumors, defined as faint, incomplete membrane staining in ≤10% of tumor cells (i.e. IHC >0 and <1+), similar numerical improvements in progression-free survival and OS were observed favoring T-DXd. Although the study only enrolled patients with hormone receptor-positive breast cancer, it emphasized the importance of HER2 expression as an actionable therapeutic target. DESTINY-Breast15 is a phase IIIB trial further evaluating the activity of T-DXd in patients with advanced HER2-low or HER2 IHC score 0 breast cancer, including hormone receptor-negative (i.e. TNBC) tumors (NCT05950945).

With the approval of T-DXd for advanced HER2-low breast cancer and emerging data in patients with HER2-ultralow tumors, optimizing pathology reports for HER2 testing in HER2-negative breast cancer is key. A refined report integrating IHC and ISH techniques in line with the 2023 ASCO/CAP updates and 2023 European Society for Medical Oncology consensus statements, along with detailed documentation of scoring the percentage (≤10%) of immunostained cells in samples with an IHC score of 0, methodology, and key technical aspects like antibody use was suggested by Ivanova et al.^30^ to standardize pathology reporting. Clear and precise HER2 status reporting is essential to guide treatment decisions and personalized patient care. Viale et al.^31^ highlighted the importance of standardized pathologist training for accurate HER2 status identification based on a global, multicenter retrospective study that suggested that almost one-third of historical IHC score 0 tumors were rescored as HER2-low on blinded reassessment, identifying potential candidates for HER2-low-directed treatments.

A significant proportion of tumors in our study exhibited discordant HER2 status following treatment, with some HER2 IHC score 0 tumors exhibiting HER2 expression according to IHC after NAT, and some HER2-low tumors demonstrating HER2 IHC score 0 status in residual tumor cells. This phenomenon underscores the dynamic nature of tumor biology and the potential impact of treatment on tumor characteristics, although it remains unclear if discordance in HER2 status is due to tumor evolution and treatment-selective pressure, analytical factors in HER2 testing, or tumor heterogeneity which could have implications for therapeutic response and long-term outcomes in patients with eTNBC. Previous studies^32^^,^^33^ suggested that HER2-positive tumors, even those with low HER2 expression, exhibit biological characteristics and response to targeted therapies distinct from those of HER2 IHC score 0 tumors. More recently, a study found no difference in outcomes by HER2 status change between HER2-low and HER2 IHC score 0.^34^ In the Dutch Pathology Registry^35^ involving 11 988 patients, about 32% had discordant HER2 status between pre- and post-NAT samples. HER2-low tumors had a slightly lower pCR rate in hormone receptor-positive cases (4% versus 5%; *P* = 0.022), although this absolute difference is relatively small. No significant survival differences were observed. In another study^36^ of 1299 patients with paired primary and recurrent/metastatic tumors, 28.5% showed HER2 conversion from primary to metastatic lesions. In the metastatic setting, patients with HER2 IHC score 0 tumors had worse OS compared to those with HER2-low metastases; however, there was no significant difference in OS by HER2 IHC when assessed in the primary tumor. Patients with HER2 IHC score 0 primary tumors that converted to HER2-low in the metastasis had longer survival than those with consistent HER2 IHC score 0 status.

Strengths of our study included the large sample size across multiple institutions and the diverse population with inclusion of 13.5% African American patients, addressing the underrepresentation seen in clinical trials. This enhances the generalizability of the findings, contributing to the generation of more relevant data for a wider population. Our study had some limitations due to its retrospective nature, absence of central testing or review of slides at the three institutions, and relatively short follow-up period (although historical data suggest that most eTNBC recurrences occur within the first 5 years from diagnosis). A key limitation of this study is the presence of incomplete data, including 16.1% of patients with unknown axillary surgery status, 2.6% with missing chemotherapy details, and 12.0% with incomplete pathology results. These data gaps could introduce bias and should be considered when interpreting the results. Also, HER2 IHC in patients with eTNBC, including HER2-low status, can be dynamic over time,^37^ potentially impacting treatment response and clinical outcomes. Central review was not carried out, and the interobserver variability between HER2 IHC score 0 and HER2-low or testing variability is well documented.^27^ Recently, investigators in the KEYNOTE-522 clinical study reported that the addition of pembrolizumab to NAT for eTNBC significantly improved pCR and event-free survival.^38^^,^^39^ We conducted this study before the addition of pembrolizumab to NAT^38^ became a standard treatment regimen for patients diagnosed with stage II-III eTNBC. Nevertheless, to the best of our knowledge, the present study represents the most extensive dataset and comprehensive analysis of HER2-low eTNBC across three academic institutions.

### Conclusions

Overall, the findings of our study contribute to a growing body of evidence that HER2-low breast cancer may not be distinct from HER2 IHC score 0 breast cancer regarding response to NAT and long-term outcomes. This has important implications for the clinical management of HER2 IHC score 0 breast cancer and highlights the need for continued research to identify more precise biomarkers that can guide treatment decisions and improve prognoses.
