# Supplementary material for: Clinical outcomes of early-stage triple-negative breast cancer after neoadjuvant chemotherapy according to HER2-low status☆
Source: ESMO Open. 2024 Nov 4;9(11):103973. doi: 10.1016/j.esmoop.2024.103973 (PMC11570474; doi:10.1016/j.esmoop.2024.103973)
Supplement: Supplemental Tables 1-4 [file mmc1.docx]

**SUPPLEMENTARY MATERIAL**

**Clinical outcomes of early-stage triple-negative breast cancer after neoadjuvant chemotherapy according to HER2-low status**

**Supplementary Table 1.** Additional patient characteristics

|  | **n (%)** | | |  |
| --- | --- | --- | --- | --- |
| **Characteristic** | **Overall**  **(n = 977)** | **HER2-low**  **(n = 388)** | **HER2 IHC score 0**  **(n = 589)** | **P-value** |
| Age at primary surgery, years | | | | .101 |
| Mean (± SD) | 51.0 ± 12.7 | 51.8 ± 12.5 | 50.5 ± 12.7 |  |
| Median (range) | 50.8 (21.5-84.0) | 51.0 (21.7-84.0) | 50.8 (21.5-84.0) |  |
| Sex | | | | 1 |
| Female | 975 (99.8) | 387 (99.7) | 588 (99.8) |  |
| Male | 2 (0.2) | 1 (0.3) | 1 (0.2) |  |
| Laterality | | | | .955 |
| Left | 511 (52.3) | 202 (52.1) | 309 (52.5) |  |
| Right | 466 (47.7) | 186 (47.9) | 280 (47.5) |  |
| T stage | | | | .812 (T0/1 vs. T2 vs. T3 vs. T4) |
| T0 | 15 (1.5) | 12 (3.1) | 3 (0.5) |  |
| T1 | 188 (19.2) | 66 (17.0) | 122 (20.7) |  |
| T2 | 522 (53.4) | 203 (52.3) | 319 (54.2) |  |
| T3 | 137 (14.0) | 57 (14.7) | 80 (13.6) |  |
| T4 | 112 (11.5) | 48 (12.4) | 64 (10.9) |  |
| TX | 3 (0.3) | 2 (0.5) | 1 (0.2) |  |
| N stage | | | | **.017** (N0 vs. N1 vs. N2 vs. N3)  **.011** (N0 vs. N+) |
| N0 | 489 (50.1) | 174 (44.8) | 315 (53.5) |  |
| N1 | 332 (34.0) | 143 (36.9) | 189 (32.1) |  |
| N2 | 65 (6.7) | 24 (6.2) | 41 (7.0) |  |
| N3 | 90 (9.2) | 46 (11.9) | 44 (7.5) |  |
| NX | 1 (0.1) | 1 (0.3) | 0 |  |
| IIIC | 94 (9.6) | 48 (12.4) | 46 (7.8) |  |
| Radiation therapy |  |  |  | .971 |
| Yes | 727 (74.4) | 288 (74.2) | 439 (74.5) |  |
| No | 248 (25.4) | 99 (25.5) | 149 (25.3) |  |
| Unknown | 2 (0.2) | 1 (0.3) | 1 (0.2) |  |

**Supplementary Table 2.** Odds ratio of pCR rate by HER2 expression with and without adjustment for clinical factors

| **Clinical Factors** | **Odds ratio, HER2-low vs. HER2 IHC score 0**  **(95% CI)** | **P-value** |
| --- | --- | --- |
| Unadjusted | 0.97 (0.74-1.28) | .835 |
| Adjusted^a^ | 0.99 (0.74-1.32) | .924 |

^a^ Adjusted factors: age at diagnosis, race, anatomic clinical stage, gBRCA status, histology, HR status, and anthracycline- and taxane-based NAT.

**Supplementary Table 3.** pCR rates and RCB following NAT compared between HER2-low and HER2 IHC score 0 status groups.

|  | **n (%)** | | |  |
| --- | --- | --- | --- | --- |
| **Parameter** | **Total**  **(n = 791)** | **HER2-low**  **(n = 324)** | **HER2 IHC score 0**  **(n = 467)** | **P-value** |
| pCR | 264 (33.4) | 100 (30.9) | 164 (35.1) | .242 |
| RD | 527 (66.6) | 224 (69.1) | 303 (64.9) |  |
| RCB 0 | 264 (33.4) | 100 (30.9) | 164 (35.1) | .128 |
| RCB I | 78 (9.9) | 33 (10.2) | 45 (9.6) |  |
| RCB II | 239 (30.2) | 90 (27.8) | 149 (31.9) |  |
| RCB III | 108 (13.6) | 54 (16.6) | 54 (11.6) |  |
| Unknown/not documented. | 24 (3.0) | 8 (2.5) | 16 (3.4) |  |

pCR = Pathological Complete Response; RD = Residual Disease; RCB = Residual Cancer Burden.

**Supplementary Table 4.** NAT outcomes based on g*BRCA* status and HER2 expression.

|  | **n (%)** | | |  |
| --- | --- | --- | --- | --- |
| **Parameter** | **Total**  **(n = 780)** | **g*BRCA* mutation**  **(n = 142)** | **g*BRCA* wild-type**  **(n = 638)** | **P-value** |
| pCR | 255 (32.7) | 63 (44.4) | 192 (30.1) | **.001** |
| RD | 525 (67.3) | 79 (55.6) | 446 (69.9) |  |
| RCB 0 | 255 (32.7) | 63 (44.4) | 192 (30.1) | **.012** |
| RCB I | 73 (9.4) | 9 (6.3) | 64 (10.0) |  |
| RCB II | 252 (32.3) | 43 (30.3) | 209 (32.7) |  |
| RCB III | 116 (14.9) | 15 (10.6) | 101 (15.9) |  |
| Unknown/not documented. | 23 (2.9) | 3 (2.1) | 20 (3.1) |  |
| HER2-low | 298 (38.2) | 50 (35.2) | 248 (38.9) | .474 |
| HER2 IHC score 0 | 482 (61.8) | 92 (64.8) | 390 (61.1) |  |
| HER2 status conversion | n = 293 | n = 47 | n = 246 | 1 |
| Yes | 96 (32.8) | 15 (31.9) | 81 (32.9) |  |
| No | 197 (67.2) | 32 (68.1) | 165 (67.1) |  |
| A+T NAT | 633 (81.2) | 87 (61.3) | 546 (85.6) | **<.001** |
| non- A+T NAT | 147 (18.8) | 55 (38.7) | 92 (14.4) |  |

pCR = Pathological Complete Response; RD = Residual Disease.

RCB = Residual Cancer Burden; HER2 status conversion = changes in the HER2 status of a tumor.

A+T = Anthracycline- and taxane-based
